# Skeletal muscle DNA methylation: Effects of exercise and HIV

**DOI:** 10.1111/acel.14025

**Published:** 2023-11-03

**Authors:** Catherine M. Jankowski, Iain R. Konigsberg, Melissa P. Wilson, Jing Sun, Todd T. Brown, Colleen G. Julian, Kristine M. Erlandson

**Affiliations:** ^1^ College of Nursing University of Colorado Anschutz Medical Campus Colorado Aurora USA; ^2^ Department of Biomedical Informatics University of Colorado Anschutz Medical Campus Colorado Aurora USA; ^3^ Division of Infectious Diseases, Department of Medicine University of Colorado Anschutz Medical Campus Colorado Aurora USA; ^4^ Department of Epidemiology Johns Hopkins Bloomberg School of Public Health Maryland Baltimore USA; ^5^ Division of Endocrinology, Diabetes, & Metabolism, Department of Medicine Johns Hopkins University Maryland Baltimore USA; ^6^ Division of Geriatric Medicine, Department of Medicine University of Colorado Anschutz Medical Campus Colorado Aurora USA

**Keywords:** aging, DNA methylation, epigenetic, epigenetic clock, exercise training, HIV, skeletal muscle

## Abstract

Aging, human immunodeficiency virus (HIV) infection, and antiretroviral therapy modify the epigenetic profile and function of cells and tissues, including skeletal muscle (SkM). In some cells, accelerated epigenetic aging begins very soon after the initial HIV infection, potentially setting the stage for the early onset of frailty. Exercise imparts epigenetic modifications in SkM that may underpin some health benefits, including delayed frailty, in people living with HIV (PWH). In this first report of exercise‐related changes in SkM DNA methylation among PWH, we investigated the impact of 24 weeks of aerobic and resistance exercise training on SkM (vastus lateralis) DNA methylation profiles and epigenetic age acceleration (EAA) in older, virally suppressed PWH (*n* = 12) and uninfected controls (*n* = 18), and associations of EAA with physical function at baseline. We identified 983 differentially methylated positions (DMPs) in PWH and controls at baseline and 237 DMPs after training. The influence of HIV serostatus on SkM methylation was more pronounced than that of exercise training. There was little overlap in the genes associated with the probes most significantly differentiated by exercise training within each group. Baseline EAA (mean ± SD) was similar between PWH (−0.4 ± 2.5 years) and controls (0.2 ± 2.6 years), and the exercise effect was not significant (*p* = 0.79). EAA and physical function at baseline were not significantly correlated (all *p* ≥ 0.10). This preliminary investigation suggests HIV‐specific epigenetic adaptations in SkM with exercise training but confirmation in a larger study that includes transcriptomic analysis is warranted.

## Graphical abstract

Accelerated epigenetic aging occurs in circulating cells in persons with HIV, a population at risk of frailty. Exercise may impart epigenetic modifications in skeletal muscle (SKm) that benefit PWH, and the general population, as they age. In this preliminary study of older adults with or without HIV, we found that SkM DNA methylation was impacted more by HIV serostatus than 24 weeks of aerobic and resistance exercise training, with some evidence suggesting HIV‐specific modifications of genes and related biological pathways. Graphic Created with BioRender.com.

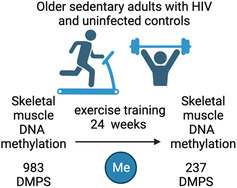

**Abbreviations**
1‐RM: one‐repetition maximum
ALS: amyotrophic lateral sclerosis
ART: antiretroviral therapy
BMI: body mass index
CpG: cytosine guanine
DIN: DNA integrity number
DMPs: differentially methylated positions
DMRs: Differentially methylated regions
DNAm age: SkM methylation age
EAA: epigenetic age acceleration
FDR: False discovery rate
GAS: γ‐interferon‐activated site
GDNF: glial cell‐derived neurotrophic factor
HIV: human immunodeficiency virus
IPA: ingenuity pathway analysis
PCA: principal component analysis
PWH: people with HIV
SkM: skeletal muscle
TSS: transcription start site
VO_2_ peak: peak aerobic capacity
YAP: yes‐associated protein

## 1 INTRODUCTION

People with HIV (PWH) are living longer due in part to effective combination antiretroviral therapy (ART). In 2018, over half (51%) of PWH in the United States were aged 50 and older (Centers for Disease Control and Prevention, 2018). Compared to HIV‐uninfected populations, PWH receiving effective ART have excess morbidity and mortality (Effros et al., 2008), earlier occurrence of aging complications including diabetes, hypertension, cardiovascular disease, osteoporosis, and fractures (Womack et al., 2011), and are at risk for early‐onset frailty or frailty‐like syndromes (Oursler et al., 2011). PWH also experience greater than expected impairments in key components of daily function including walking speed, balance, ability to rise from a chair, and peak aerobic capacity (Oursler et al., 2006). The clinical importance of functional decline cannot be underestimated; HIV infection with impaired physical function increases mortality risk beyond HIV infection or impaired function alone (Greene et al., 2014).

While the molecular mechanisms underpinning impaired physical function in older PWH are not well defined, likely contributors include chronic inflammation and immune activation (Erlandson et al., 2013) and mitochondrial dysfunction related to HIV and ART exposure (Schank et al., 2021). We found that exercise training improved peak aerobic capacity and physical function in older PWH (Erlandson et al., 2018) but that some mitochondrial adaptations in skeletal muscle were blunted compared to uninfected controls (Jankowski, Wilson, et al., 2020). Elucidating the mechanisms underlying the benefits of exercise training in PWH may reveal candidate biological pathways that could be the targets of novel preventative or therapeutic approaches that reduce the burden of physical impairment associated with HIV infection. More broadly, HIV infection represents a once lethal but now survivable chronic condition affecting many of the cellular, molecular, and compensatory systems associated with aging (Lopez‐Otin & Kroemer, 2021). It is therefore also possible that while aging with chronic HIV infection (a long‐term stressor), alternative mechanistic pathways in cells and tissues are activated and integrated in response to exercise training (repetitive acute stressor), suggesting biological resilience (Hadley et al., 2017).

Epigenetic modifications in skeletal muscle (SkM) may explain some of the metabolic and functional impairments found in PWH. Epigenetic marks are cell‐heritable genetic modifiers that do not alter the underlying DNA sequence but can modify gene expression and are responsive to aging, environmental stimuli, and disease (Jirtle & Skinner, 2007). Several distinct epigenetic mechanisms exist, including DNA methylation, histone modification, histone variants, and RNA‐based mechanisms. Here, we focus on genome‐wide DNA methylation, defined by the addition of a methyl group to the C‐5 position of cytosine residues within cytosine‐guanine (CpG) dinucleotides, given its prominent role in transcriptional regulation and previous evidence that DNA methylation status of SkM is altered by age (Voisin et al., 2021) and exercise training (Fiorito et al., 2021; Gorski et al., 2023; Lindholm et al., 2014; Rowlands et al., 2014; Voisin et al., 2023). In middle‐aged adults without HIV infection, aerobic endurance (Fiorito et al., 2021; Rowlands et al., 2014) or resistance (Gorski et al., 2023; Rowlands et al., 2014) exercise training caused changes in SkM DNA methylation. Furthermore, in a meta‐analysis of six independent cohorts, exercise training resulted in a shift toward a younger SkM DNA methylome and transcriptome in older adults. (Voisin et al., 2023).

However, to our knowledge, no study has investigated how exercise training affects SkM DNA methylation in PWH or whether the methylation responses differ from those of HIV‐uninfected people of similar chronological age.

Epigenetic clocks make use of algorithms based on the DNA methylation of specific CpG sites in cells or tissues that are strongly correlated to chronological age (Bell et al., 2019). Variance between DNA methylation age (DNAm age) and chronological age is thought to encapsulate biological aging as defined by physiological biomarkers or adverse health outcomes (Bell et al., 2019). Epigenetic age acceleration (EAA) is expressed as the residual resulting from the regression of DNAm age with chronological age (Levine et al., 2018). The initial HIV infection has been reported to accelerate the epigenetic age of peripheral blood mononuclear cells (Breen et al., 2022), and ART partially reverses this acceleration (Esteban‐Cantos et al., 2021). Indicating pronounced biological age acceleration with HIV infection, the EAA of peripheral blood mononuclear cells was approximately 8 years (e.g., biological age > chronological age) in ART‐naïve PWH (Sehl et al., 2020) and 6 years after 2 years of ART (Sehl et al., 2020). A muscle‐specific epigenetic clock, MEAT V2.0, (Voisin et al., 2020) accurately predicts chronological age from the methylation of 156 CpG sites. Thus, further investigation is needed to determine if EAA is found in SkM of aged PWH when using a muscle‐specific epigenetic clock.

**FIGURE 1:**
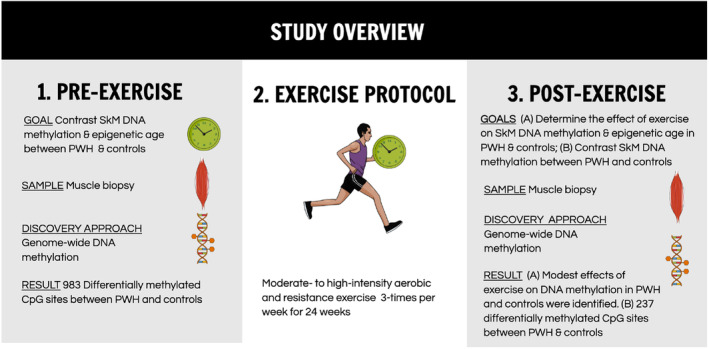
Exercise for healthy aging epigenetic sub‐study overview. PWH, people with HIV.

In the Exercise for Healthy Aging Study, we found that middle‐aged PWH had worse baseline physical function than age‐matched uninfected controls (Erlandson et al., 2018). In response to 24 weeks of aerobic endurance and resistance exercise training; however, PWH had greater relative improvements in peak aerobic capacity (VO_2_ peak) and 400‐m walk time than uninfected controls (Erlandson et al., 2018), yet smaller increases in lean mass (Jankowski, Mawhinney, et al., 2020) and blunted changes in markers of SkM mitochondrial respiration (e.g., citrate synthase activity) (Jankowski, Wilson, et al., 2020). These functional and molecular differences lead to the current preliminary investigation of SkM DNA methylation profiles and EAA in PWH and uninfected controls. Specifically, we aimed to (1) examine whole‐genome DNA methylation profiles in middle‐aged PWH and uninfected controls at baseline (pretraining) and after exercise intervention; (2) determine changes in SkM DNA methylation profiles with exercise training in PWH and uninfected controls; (3) using MEAT V2.0, compare SkM EAA in PWH and uninfected controls at baseline and after training; and (4) test for associations between SkM EAA and physical function before exercise training (Figure 1).

## 2 METHODS

### 2.1 Participants

The Exercise for Healthy Aging Study enrolled PWH and HIV‐uninfected controls (“controls”) from April 2014 to May 2017 (Clinical Trials NCT02404792). The inclusion and exclusion criteria and study design of the Exercise for Healthy Aging Study have been published (Erlandson et al., 2018). Briefly, at study entry the participants were aged 50–75 years, sedentary (<60 min of physical activity per week for the preceding 6 months by self‐report), had a body mass index (BMI) between 20 and 40 kg/m^2^, had no contraindications to exercise, and did not use intramuscular testosterone (stable use of physiological doses of transdermal testosterone was permitted). Comorbidities and use of statins were self‐reported and confirmed by the review of electronic health records. All PWH included in this study were on stable ART with undetectable HIV‐1 RNA, as defined by <20 copies/mL, for at least 2 years and had CD4+ T‐cell count above 200 cells/μL. Our current analysis was limited to men (*n* = 30) in the Exercise for Healthy Aging Study because only one woman consented to a muscle biopsy. All study procedures were approved by the Colorado Multiple Institution Review Board (IRB 14‐2207). Written informed consent was obtained from all participants.

### 2.2 Exercise intervention and physical function assessments

Participants completed three supervised exercise sessions each week for 24 weeks at the University of Colorado‐Anschutz Medical Campus Exercise Research Laboratory. As previously described (Erlandson et al., 2018), VO_2_ peak was measured prior to training and at Weeks 12 and 24 using a graded treadmill test. Maximum muscle strength of the upper (bench press) and lower body (leg press) was measured using the one‐repetition maximum (1‐RM; maximum weight that can be lifted only one time) at baseline and every 3 weeks up to Week 24; ten‐repetition chair rise time was measured before and after 24 weeks of training.

Each exercise session included walking or jogging on a treadmill and weight‐assisted machine (Cybex International) resistance exercises (bench press, leg press, lateral pulldown, and a rotating fourth exercise). Total exercise time gradually increased to a target of 50 min per session. The first 12 weeks targeted moderate‐intensity aerobic (40%–50% VO_2_ peak) and resistance exercises (60%–70% 1‐RM, 3 sets of 8 repetitions) during each session. At week 12, participants were randomized to continue moderate‐intensity training or advance to high‐intensity training (60%–70% of week 12 VO_2_ peak and >80% 1‐RM) (Erlandson et al., 2018). The exercise training protocol was designed to match the 2008 Department of Health and Human Services recommendations for physical activity in adults (DHHS, 2008). The high‐intensity arm (“vigorous” per DHHS recommendations) was to preliminarily evaluate whether higher intensity exercise conferred benefits beyond that of moderate‐intensity exercise (Erlandson et al., 2018).

To minimize the influence of daily physical activity or dietary changes on study outcomes, participants were asked to maintain their usual physical activity levels (other than the supervised training sessions) and dietary intake over the study period.

### 2.3 Tissue biopsy and processing

Vastus lateralis specimens were obtained by percutaneous needle biopsy before and after 24 weeks of exercise training; participants fasted overnight and abstained from exercise for 24 h prior to the biopsy (Jankowski, Wilson, et al., 2020). Specimens were cleaned of visible fat, immediately immersed in liquid nitrogen, and then stored at −80°C until analysis. SkM genomic DNA was extracted using the AllPrep DNA/RNA Mini Kit (Qiagen) and assessed for quantity and integrity using the NanoDrop ND‐100 UV–Vis spectrophotometer (NanoDrop Technologies) and DNA chips on the Agilent 2100 Bioanalyzer. Integrity for all samples was established as DNA Integrity Number (DIN) greater than or equal to 8.

### 2.4 DNA methylation analysis

Genome‐wide SkM DNA methylation pre‐ and postexercise training was quantified on MethylationEPIC BeadChip arrays (Illumina) per the Illumina Infinium methylation protocol. Samples were processed as a single batch at the University of Colorado Genomics Core with PWH and control samples randomized across arrays. For a given subject, pre‐ and post‐training samples were processed on the same array to minimize the potential influence of between‐array variation on our results. Briefly, genomic DNA was bisulfite converted using the EZ DNA Methylation Gold Kit (Zymo Research), amplified and enzymatically fragmented. Bisulfite‐converted DNA was hybridized to the BeadChip array, which was subsequently scanned using the Illumina iScan system. Signal intensities of methylated and unmethylated probes were exported from the GenomeStudio interface, along with detection *p*‐values representing the likelihood of detection relative to background. Individual data points with a *p*‐value outside of detection criteria (*p* > 0.05) were treated as missing data. Data were normalized within R using SeSAMe (Zhou et al., 2018), and *M*‐values were calculated. Probes with high detection *p*‐values, poor or multiple mapping, and underlying common SNPs were removed. Batch effects were adjusted for using ComBat in the R package sva. Using limma (Smyth, 2004) we fit linear models with DNA methylation (*M* value) as the outcome variable, HIV serostatus or timepoint as the dependent variable, and the covariates chronological age and race. *p*‐Values were adjusted for bias and inflation inherent to methylome‐wide association studies through the bacon R package (van Iterson et al., 2017). Differentially methylated positions (DMPs) were defined using a false discovery rate (FDR) corrected *p*‐value < 0.05.

We first contrasted SkM DNAm profiles by HIV serostatus at baseline and postexercise intervention. This permitted us to identify the differential methylation status of PWH compared to controls prior to the exercise intervention and those that differed by serostatus after 24 weeks of exercise training. We then contrasted baseline DNA methylation profiles to post‐training profiles in PWH, uninfected controls, and the combined cohort (PWH and uninfected controls). Considering the modest sample size, we did not test for the interaction of HIV serostatus and exercise training. Principal component analysis (PCA) plots of methylation data were generated to visualize the degree to which SkM DNAm clustered by HIV serostatus and exercise training. Comparisons of PCs by diagnosis (HIV‐infected or not) and training (pre‐ vs. post‐training) were tested using *t*‐tests. In the combined cohort, no significant DMPs were identified as a function of exercise training; for this reason, we also sought to identify differentially methylated regions (DMRs) between pre‐ and postexercise intervention in PWH and controls using Comb‐p which is an analytical tool developed to spatially analyze correlated *p*‐values (Pedersen et al., 2012). The *p*‐values generated from the linear models described above were used for Comb‐p analysis. DMPs and DMRs were annotated to the nearest gene, CpG island, and other genomic features using the CruzDB tool.

Formal pathway enrichment analysis of DMPs was performed using the missMethyl R package (Phipson et al., 2016). Results are not reported as no pathways were significantly enriched. Instead, we discuss key biological pathways of potential relevance considering the genes with methylation that significantly differed by HIV serostatus or exercise. Biological pathway membership of differentially methylated genes (Barabasi et al., 2011) was performed using Ingenuity Pathway Analysis (IPA; Ingenuity Systems) software.

To determine whether our findings agree with other datasets, we contrasted pre‐ versus postexercise training (12 weeks, HIIT training) skeletal muscle DNA methylation using publicly available Illumina MethylationEPIC BeadChip data for 15 men (Voisin et al., 2020, 2021, 2023; GSE171140). As was done for our dataset, we ran linear models for each probe testing for the effect of pre‐post intervention while adjusting for individual subject ID, and bacon‐corrected the test statistics. Additionally, we compared our significant DMP lists to that of the “Epigenetics in Training” (EpiTrain) study (Lindholm et al., 2014) of 23 young women (*n* = 11) and men (*n* = 12) who performed one‐legged resistance exercise for 3 months.

### 2.5 Skeletal muscle epigenetic age acceleration analysis

The Muscle Epigenetic Age Test (MEAT v2.0) R package was used to estimate SkM methylation age (DNAm age) and EAA defined as the residuals of a linear regression of predicted age against chronological age (Voisin et al., 2021). This SkM‐specific epigenetic clock, using DNA methylation levels at 156 CpG sites across the genome, improves upon pan‐tissue epigenetic clocks by reducing the median absolute error of prediction of chronological age from 12 years to 4.4 years (Voisin et al., 2021). SkM EAA estimates were compared between PWH and controls at baseline and after exercise training, and the association of baseline physical function and SkM EAA was tested.

### 2.6 Statistical analyses

Baseline characteristics were compared via *t*‐tests, Mann–Whitney, or Fisher's exact tests. Exercise training adherence was calculated as the percent of the 72 total exercise sessions attended (median, IQR). Differences in SkM EAA were analyzed using a mixed‐effects (group × training) model. The associations of baseline SkM EAA with physical function at baseline was tested using Pearson tests (*r*) with an alpha level of 0.05.

## 3 RESULTS

### 3.1 Participant characteristics and exercise adherence

Baseline muscle biopsies were obtained from 12 PWH and 18 uninfected controls (Table 1). PWH were of similar chronological age as controls but had significantly lower percent body fat, slower 400‐m walk time, less lower body strength, and more frequent cannabis use. PWH tended to have a lower BMI and more often reported smoking tobacco. Among PWH, all had an HIV‐1 RNA < 20 copies/mL and the median CD4 count was >500 cells/uL. The median years since HIV diagnosis and years of ART use were nearly two decades, and most PWH had prior exposure to thymidine analogues. Adherence to exercise training was 90% (86, 92%) in PWH and 84% (81, 89%) in controls (*p* = 0.18). Post‐training muscle biopsies were obtained from 10 of 12 PWH and 15 of 18 controls. Of the participants with post‐training biopsies, 6 PWH and 7 controls were randomized to continue with moderate‐intensity exercise during Weeks 13–24, whereas 4 PWH and 8 controls were randomized to high‐intensity exercise.

**TABLE 1:** Participant characteristics.

Variables	PWH, *n* = 12	Controls, *n* = 18	*p*‐Value
Male, %	100	100	
Race			
White, %	67	94	0.07
Black, %	25	6	
More than one race, %	8	0	
Ethnicity			
Non‐Hispanic, %	83	83	0.57
Hispanic, %	8	17	
Unknown/not reported, %	8	0	
Age, years	56 ± 4	57 ± 7	0.49
BMI, kg/m^2^	25.3 ± 2.6	28.5 ± 5.1	0.06
Body fat, %	22.9 ± 4.1	28.5 ± 7.2	0.02
Lean body mass, kg	57.3 ± 5.8	61.6 ± 8.0	0.12
Current tobacco smoker, %	42	11	0.08
Current cannabis use, %	67	22	0.04
Alcohol use,^a^ %	0	22	0.13
Comorbidities,^b^ ≥3, %	75	39	0.18
Current statin use, %	50	22	0.24
Current testosterone^c^ use, %	17	0	0.15
VO_2peak_, mL/kg/min	26.6 ± 4.1	29.8 ± 6.8	0.15
400‐m walk, s	252.4 ± 32.6	226.3 ± 23.5	0.02
Chair rise time, s	21.0 ± 6.6	18.1 ± 4.4	0.15
Leg press strength,^d^ kg	124.6 ± 30.1	150.8 ± 24.0	0.01
Bench press strength,^d^ kg	54.2 ± 11.6	57.2 ± 8.4	0.39
Years since HIV diagnosis	21 (18, 24)	—	
CD4 count, cells/μL	518 (403, 837)	—	
ART use, years	17 (13, 19)	—	
Thymidine analogue^e^ use, %	67	—	

*Note*: Continuous variables expressed as mean ± SD or median (IQR); comparisons made via *t*‐tests, Mann–Whitney, or Fisher's Exact Tests.

Abbreviations: ART, antiretroviral therapy; PWH, people with HIV; VO_2peak_, greatest volume of oxygen consumption achieved during a graded exercise test.

^a^ >2 drinks/day.

^b^ Hypertension, hyperlipidemia, diabetes, cardiovascular disease, or depression/anxiety/bipolar disorder (individually or in combination).

^c^ Physiological doses of testosterone for ≥3 months prior to study entry.

^d^ One‐repetition maximal strength.

^e^ Prior use >1 year.

### 3.2 DNA methylation by HIV serostatus

**FIGURE 2:**
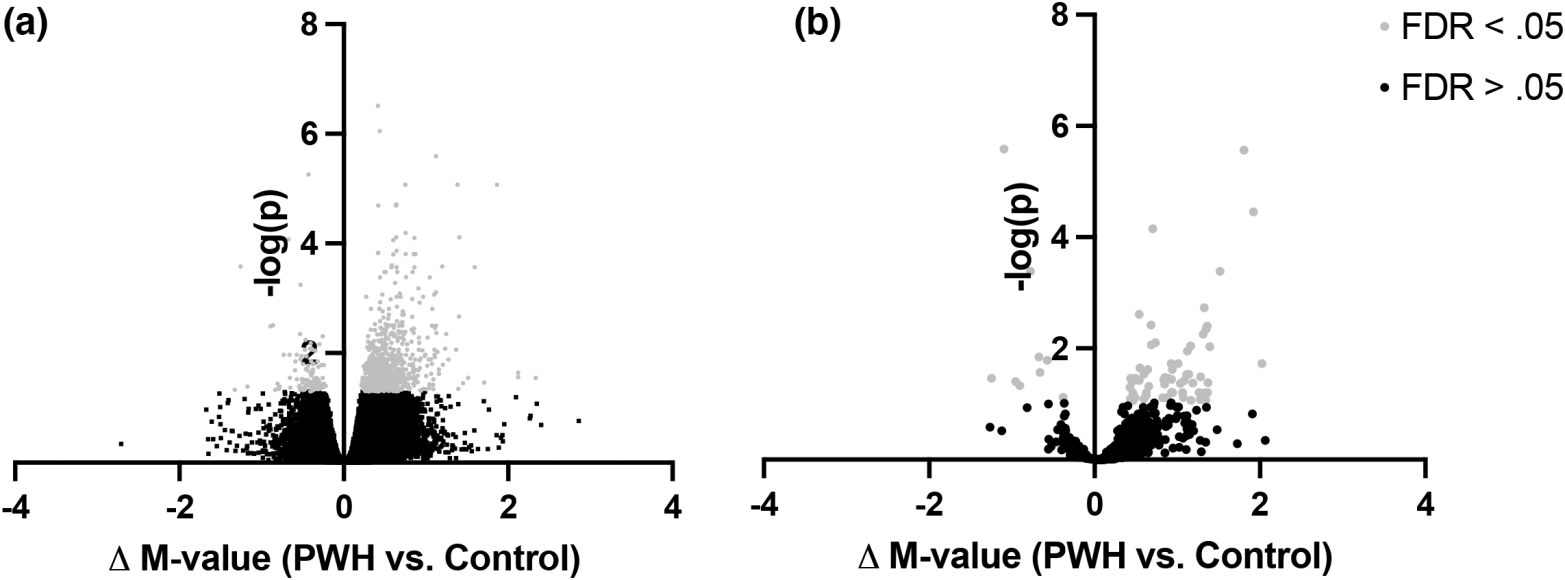
Volcano plots of differentially methylated CpG sites in skeletal muscle of people with HIV (PWH) and controls at (a) pre‐training (i.e., baseline) and (b) post‐training. Significant differentiation, gray; nonsignificant, black. FDR, false discovery rate.

We first analyzed whole‐genome DNA methylation using the MethylationEPIC BeadChip array (Illumina) to establish baseline SkM DNA methylation differences by HIV serostatus. At baseline, the SkM of PWH demonstrated 983 differentially methylated CpG sites compared to uninfected controls (Figure 2; Table S1). Based on the CpG sites available for query on the Illumina BeadChip array, PWH showed a more hypermethylated SkM profile, with 88.5% of sites being hypermethylated and 11.5% hypomethylated compared to controls. Nearly half (46%) of all DMPs (*n* = 455) annotated to or proximal to CpG islands; this proportion did not differ from that of all probes on the array (44.3%, *p* = 0.23). The 983 DMPs that differed by HIV status at baseline were depleted for promoter‐annotated probes (5.3% vs. 14.4% of all probes on the array, *p* = 6.2 × 10^−16^).

Differential methylation analysis identified DMPs (FDR adjusted *p*‐value < 0.05) associated with HIV serostatus at baseline, with the top DMP by significance level located in the 5′ UTR of the basic helix–loop–helix ARNT Like 1 (*BMAL1* or *ARNTL*) gene (Table S1). Biological pathways represented by DMPs that differed by HIV serostatus included γ‐interferon‐activated site (GAS) signaling, IL‐1 signaling, and androgen signaling.

Fewer differentially methylated CpG sites were identified between PWH and controls (68% hyper‐ and 32% hypomethylated sites) after exercise training (Figure 2; Table S2). Most (68%) of the 237 differentially methylated CpG sites (*n* = 160) annotated to or proximal to CpG islands; this proportion was significantly different than the array (44%; *p* = 1.13 × 10^−12^). The DMPs that differed after exercise training were depleted for promoter regions (6.8% vs. 14.4% of all probes; *p* = 0.001).

### 3.3 Exercise‐induced SkM DNA methylation changes stratified by HIV serostatus

**FIGURE 3:**
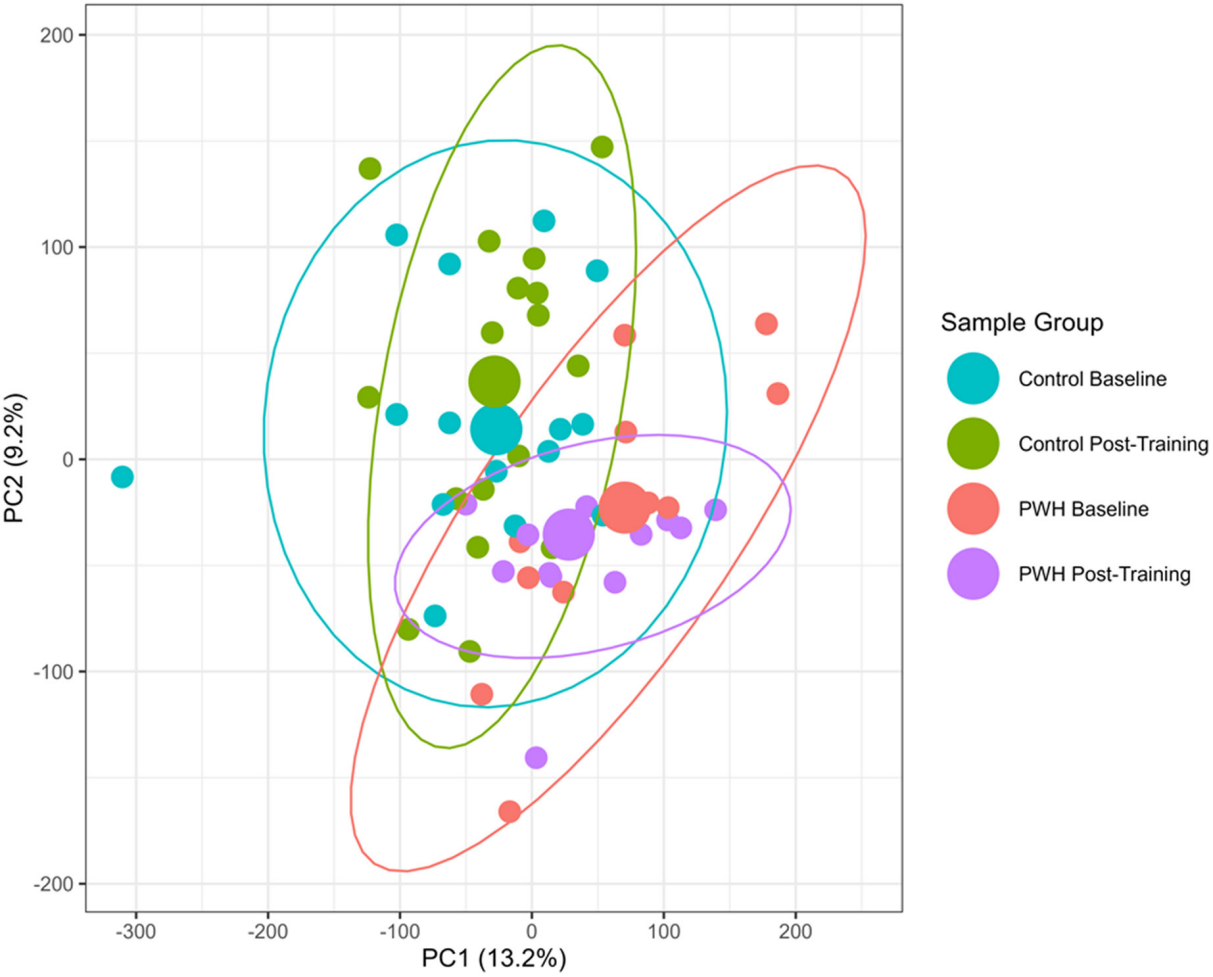
Principal component analysis (PCA) plots of SkM DNA methylation (DNAm) for people with (PWH) and uninfected controls pre‐ and postexercise training. The PCA plots show distinct clustering by HIV serostatus and minimal clustering by exercise training. Each dot represents a unique individual. PWH are designated by red (baseline) or purple (postexercise training) dots. Uninfected controls are designated by blue (baseline) or green (postexercise training) dots. Enlarged points represent the median centroid for each group.

Using PCA plots, (Figure 3) our data showed distinct clustering of DNAm by HIV serostatus. In PC1, HIV serostatus was a significant source of variation in the pre‐training, post‐training, and combined data (all, *p* < 0.01). In PC2, HIV serostatus was a significant factor in the pre‐training and combined data (*p* < 0.001), but not the post‐training data (*p* = 0.07). In contrast, training status (pre‐ or post‐intervention) was not a significant source of variation within PWH or the uninfected control cohort (all, *p* > 0.05). However, among PWH, the pre‐ and post‐training comparison identified 967 DMPs (Table S3) whereas, among uninfected controls, training resulted in 158 DMPs (Table S4).

Regional methylation analysis identified 209 DMRs (Sidak *p*‐value < 0.05) associated with exercise training in PWH, with the top DMR by significance level being located within the acid‐ sensing ion channel subunit family member 4 (*ASIC4*) gene, consisting of 7 CpG sites (Sidak = 4.54 × 10^−19^; Table 2; Table S5). In PWH, several of the DMRs identified were annotated to genes that are involved in biological pathways of potential importance for HIV or exercise adaptation, including amyotrophic lateral sclerosis signaling (ALS); glutamate receptor signaling; and glial cell‐derived neurotrophic factor (GDNF). In controls, regional methylation analysis identified 74 DMRs associated with exercise training, with the top DMR by significance level being located within the S100 calcium‐binding protein A13 (*S100A13*) gene, consisting of 13 CpG sites (Sidak = 1.44 × 10^−7^; Table 2; Table S6). In controls, several of the DMRs were annotated to genes involved with key biological pathways, including Hippo signaling, that are known to be modified by exercise training.

**TABLE 2:** Differentially methylated regions in PWH and uninfected controls pre‐and postexercise intervention.

Chr: start–end	Probes	Sidak *p*‐value	Gene
*People with HIV*			
2: 220378996–220379199	7	4.54E‐19	*ASIC4*
2: 177014555–177015125	11	1.62E‐12	*MIR10B*
12: 3862221–3862597	11	1.61E‐11	*CRACR2A*
2: 176988284–176988939	6	1.85E‐11	*HOXD9*
2: 177012117–177012706	7	3.57E‐11	*MIR10B*
2: 177028606–177028804	6	1.16E‐10	*HOXD3*
2: 177015812–177016978	12	1.26E‐10	*HOXD4*
4: 5021111–5021311	5	2.50E‐10	*CYTL1*
11: 34460107–34460789	13	1.12E‐09	*CAT*
12: 3365474–3365835	5	1.53E‐09	*TSPAN9*
1: 226926808–226927217	8	3.59E‐09	*ITPKB*
6: 106545667–106546824	13	7.63E‐08	*PRDM1*
16: 68001727–68002372	7	1.13E‐07	*SLC12A4*
12: 109181397–109182032	6	1.32E‐07	*SSH1*
12: 117798748–117799370	4	5.70E‐07	*NOS1*
*Controls*			
1: 153599479–153600064	13	1.44E‐07	*S100A13*
1: 153599479–153600064	13	1.44E‐07	*S100A13*
3: 141087119–141087363	5	2.36E‐06	*ZBTB38*
12: 122234980–122235309	4	7.26E‐06	*LINC01089*
6: 32055370–32055738	12	1.09E‐05	*TNXB*
22: 38071455–38071677	7	2.64E‐05	*LGALS1*
8: 145027741–145028170	6	3.88E‐05	*PLEC*
18: 13610941–13611576	12	4.38E‐05	*LDLRAD4;MIR4526*
6: 33048254–33048732	12	5.60E‐05	*HLA‐DPA1;HLA‐DPB1*
19: 36643532–36643771	4	8.06E‐05	*COX7A1*
161100122–161100672	6	8.27E‐05	*LPA*
7: 38350921–38351154	3	0.0001019	*TRG‐AS1*
6: 49681178–49681391	8	0.0002603	*CRISP2*
7: 27170241–27170554	7	0.0003561	*HOXA4*
1: 21586831–21587174	4	0.0004814	*ECE1*
3: 114343654–114344019	6	0.0005134	*ZBTB20*

*Note*: For ease of presentation, shown are DMRs containing 3 or more CpG sites and a Sidak < 0.01; the top 15 DMRs are presented. Complete data can be found in Table S6.

Only collagen type IV alpha 1 chain (*COL4A1*) overlapped between the exercise‐associated DMR lists of PWH and uninfected controls.

### 3.4 Contrasts with other SkM DNA methylation datasets

In the Voisin exercise training data (Voisin et al., 2020, 2021, 2023; GSE171140), we identified 86 probes significantly associated with exercise intervention (Table S7). No individual probes overlapped between those identified in the Voisin dataset and those identified in our control or PWH cohort. We then compared genes represented by DMPs identified in the Voisin data set and our control or PWH cohorts. Between our analysis in controls and Voisin, we get overlap of a single gene: RP11‐242B12.5. In our data, we see cg13453485 in the 5′ UTR hypomethylated post‐intervention. In Voisin's data, we found cg08129312 hypomethylated post‐intervention, and it is also in the 5′ UTR. Between our analysis in PWH and Voisin, we get overlap of 2 genes: U6 and TEKT1. In our analysis, we find hypomethylation of cg16858509 in the body of U6 and hypomethylation of cg24871068 within 1500 bases of the transcription start site (TSS) of TEKT1. In the Voisin dataset, we found hypomethylation of cg07087737 within 1500 bases of the TSS of U6 and hypomethylation of cg12685753 within 1500 bases of the TSS of TEKT1.

In the comparison of our significant DMP lists to that of the EpiTrain study (Lindholm et al., 2014), we found that 22 sites overlapped between their list of DMPs and our HIV list (although directionality did not always agree), and two sites that overlapped between their DMP list and our control list (Table S8).

### 3.5 SkM epigenetic age acceleration by HIV serostatus and training

**FIGURE 4:**
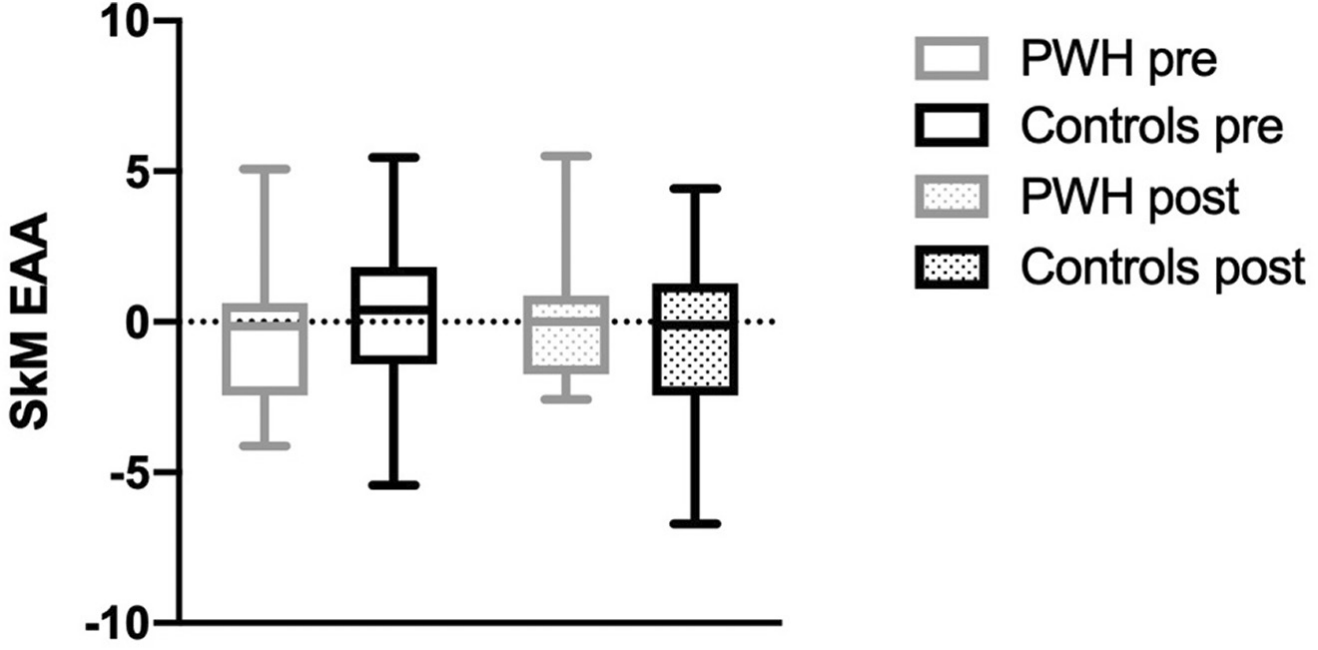
Skeletal muscle epigenetic age acceleration (SkM EAA) pre‐ and postexercise training in PWH (*n* = 12) and controls (*n* = 18). The dotted line represents the mean chronological age (pre‐training). SkM EAA estimated using the MEAT V2.0 epigenetic clock (Voisin et al., 2021). Pre‐training comparison, *p* = 0.50; post‐training comparison, *p* = 0.79).

Using the MEAT V2.0 epigenetic clock (Voisin et al., 2021), we found that SkM EAA at baseline was not significantly different between PWH (*n* = 12) and controls (*n* = 18; *p* = 0.50; Figure 4), and the main effect of exercise training on SkM EAA was not significant (*p* = 0.79). There were no significant correlations (all *p* ≥ 0.10) between baseline SkM EAA and physical function tests (*r*‐coefficients for chair rise time, −0.19; maximum strength on bench press, 0.15 and leg press, 0.34).

## 4 DISCUSSION

To the best of our knowledge, this is the first investigation to describe SkM DNA methylation differences in older PWH and uninfected controls and test the effects of exercise training on SkM DNA methylation profiles. We found that SkM DNAm differentiated clearly on HIV serostatus with relatively modest exercise training effects. Approximately six times more DMPs were identified with exercise training in PWH compared to controls, suggesting a larger impact of training on DNA methylation in PWH although the differential effects may be partially due to differences in exercise intensity between the groups. In our study, PWH were virally suppressed with decades‐long exposure to the sequalae associated with HIV, such as chronic inflammation, and the side effects of ART. In contrast, the exercise training exposure was relatively short but presented a physiological stimulus to both groups of older, previously sedentary men. As noted by Lindholm et al. (2014), the changes in DNA methylation in response to environmental conditions (i.e., exercise) are slight when compared to disease‐related conditions (i.e., HIV infection). Nonetheless, aerobic and resistance exercise training are associated with a shift toward a “younger” methylome (Voisin et al., 2023), thus supporting that exercise training triggers some effects that oppose aging.

By way of genome‐wide analyses, we found that DMPs were hypermethylated in PWH compared to controls before and after exercise training. Antoun et al. (2022) found that in SkM of older men, 53% of DMPs distinguished by the presence of sarcopenia were hypermethylated. In their study, sarcopenia was defined as low appendicular lean mass index with low gait speed, low grip strength, or both. In survivors of breast cancer (Gorski et al., 2023), 57% of DMPs in SkM were hypermethylated compared to age‐matched women without a history of breast cancer; neither group were exercise trained. Similar to our study, exercise training was associated with more differentially methylated CpG sites in women with breast cancer compared to controls. Whether methylation results in the up‐ or downregulation of associated genes must be considered to understand the functional relevance of the modification.

At baseline, genes annotated to significant DMPs were involved in key biological pathways, such as γ‐interferon‐activated site (GAS) signaling. An activator of gene transcription, the interferon γ activation sequence of nucleotides was originally studied for its role in cytokine responses to viruses, including HIV‐1 (Sgarbanti et al., 2004), but is also involved with inflammation, cell proliferation, apoptosis, and adaptive immunity (Michalska et al., 2018), systems that are recognized as hallmarks of aging (Lopez‐Otin & Kroemer, 2021). The GAS signaling pathway may be a target for future studies that include enrichment analysis to advance understand of its functional relevance.

To evaluate the exercise training responses within each group we identified genes annotated to regions with significant exercise training responses. In PWH, the genes annotated to the DMRs were involved with ALS signaling and the GDNF pathway, among other biological pathways. Clinical and bioinformatic evidence suggests a relationship between retroviruses, including HIV, and ALS or ALS‐like disease, perhaps through common mechanisms of neuroinflammation (Cintron‐Colon et al., 2020; Verma & Berger, 2006). The GDNF pathway maintains neuron‐to‐target tissue communication, including the function of the neuromuscular junction (Cintron‐Colon et al., 2020). Aging and neuromuscular diseases such as ALS are associated with adverse changes in the morphology and function of the neuromuscular junction, whereas exercise has opposing effects (Pratt et al., 2021), potentially mediated through increased GDNF protein expression (Dobrowolny et al., 2021).

In uninfected controls, exercise modified the methylation status of several genes involved in Hippo signaling. Hippo, a regulator of cell proliferation and organ size, has been a focus of cancer research and its function in SkM has been studied in the context of exercise, aging, sarcopenia, and obesity (Setiawan et al., 2021; Turner et al., 2020). Two key Hippo effectors, Yes‐associated protein (YAP) and TAZ (a component of the WW domain‐containing protein‐1; Wwtr1), are regulated in muscle by a number of endurance and resistance exercise‐related signals (Gabriel et al., 2016) that could have been generated by the combined exercise in the Healthy Aging Study. Hippo was among the most significantly enriched pathways identified in a 2‐year, moderate‐intensity physical activity intervention in women (aged 50–69 years) without HIV infection (Fiorito et al., 2021).

To the best of our knowledge, ours is the first study to apply a muscle‐specific epigenetic clock (Voisin et al., 2021) to SkM of PWH. We pursued this approach because accelerated epigenetic aging has been associated with HIV infection in circulating cells. Phenotypically, more PWH than controls in our study were prefrail and had worse performance on physical function tests (Erlandson et al., 2018), specifically slower 400‐meter walk times and lower leg strength. However, SkM EAA was not evident in the PWH or uninfected controls at baseline or after training.

The lack of agreement between our findings and those derived from the Voisin dataset (Voisin et al., 2020, 2021, 2023) and the EpiTrain dataset (Lindholm et al., 2014) may be attributed to differences in baseline subject characteristics (e.g., health status, sex), the length and type of exercise intervention, or technical factors. The comparably small number of individuals with pre‐ and postexercise DNA methylation data in our study and the two studies used for comparison may also contribute to minimal DMP overlap. Notably, only two sites overlapped between DMPs identified in the Voisin and EpiTrain lists, further highlighting the context‐specific nature of DNA methylation.

Our observation that serostatus was not associated with SkM EAA may be due, in part, to factors inherent to the MEAT epigenetic clock. The MEAT epigenetic clock was not trained to predict biological age but rather chronological age, which is not a dependable index of the physiologic biomarkers or adverse health outcomes that reflect biological age (Levine et al., 2018; Voisin et al., 2023). Moreover, algorithms underlying epigenetic clocks designed to predict chronological age with high accuracy preferentially select CpGs strongly associated with chronological age but unaffected by other factors, including disease status, diet, or smoking (Field et al., 2018). As a result, MEAT and other epigenetic clocks do not provide refined functional readouts of biological age. Thus, variation in methylation due to HIV serostatus was selected out when using MEAT but was apparent in the genome‐wide analyses (Figure 2). In line with this rationale, no study using MEAT, including the present one, has identified associations between SkM epigenetic age and sarcopenia (Antoun et al., 2022), physical function (Sillanpaa et al., 2021) or exercise training (Gorski et al., 2023).

The strengths of our study include the focus on epigenetics in a target tissue of exercise, namely SkM, in a population that is aging with the sequelae of HIV infection (PWH) and side effects of ART, including blunting of some SkM mitochondrial responses to exercise training (Jankowski, Wilson, et al., 2020). The groups were well matched on chronological age and all participants were sedentary before joining the trial. The exercise intervention was supervised and adherence to training was high (≥84%) in PWH and controls.

While our report contributes to the understanding of epigenetic changes in muscle imparted by exercise in middle‐aged adults and is the only study of SkM DNAm in PWH to date, we also acknowledge several important limitations. The sample size was small and participants differed on key characteristics that could contribute to DNA methylation including greater adiposity (potentially attributed to ART) and use of cannabis and tobacco than controls. Although the training protocol implemented in this project was designed to match the 2008 US public health guidelines for exercise (DHHS, 2008), combining exercise modes may have masked the independent effects of either exercise mode alone (i.e., aerobic vs. strength). In the parent study (Erlandson et al., 2018), exercise intensity increased from moderate to higher for half of the participants at Week 12 of the 24‐week intervention. The distribution of PWH and controls in this analysis was balanced in the moderate‐intensity arm but PWH were under‐represented in the high‐intensity arm. Thus, the results in PWH may be most reflective of moderate‐intensity exercise, but this is a comfortable and recommended exercise intensity for health benefits. The sample size was insufficient for sub‐analyses of exercise mode‐ or intensity‐effects on SkM DNA methylation or to measure pathway enrichment. Although the number of unique individuals included in our study is equivalent to or greater than several other studies investigating the impact of exercise intervention on SkM DNAm (Garcia et al., 2022; Gorski et al., 2023; Lindholm et al., 2014), our sample size precluded the examination of potential effect modifiers such as classes of ART used in the past and during the study; however, the initial HIV infection may have the strongest effect on epigenetic aging (Breen et al., 2022; Sehl et al., 2020). In short, our findings should be considered preliminary and hypothesis‐generating. Importantly, our epigenetic analyses were confined to SkM specimens from men due to the lack of women consenting for SkM biopsy. Future studies including women are required and should also determine the variability in DNA methylation attributable to sex (Lindholm et al., 2014). Although MEAT V2.0 is the most accurate epigenetic clock algorithm for skeletal muscle, the median error of the estimate is 4.4 years (Voisin et al., 2021). We lacked the clinical data needed to apply second‐generation algorithms that were trained on age‐related or disease phenotypes in addition to chronological age, such as DNAm PhenoAge. Interestingly, exercise was associated with younger epigenetic age, and smoking with accelerated age, as measured in blood and estimated by DNAm PhenoAge (Levine et al., 2018). Finally, paired methylome and transcriptome analyses of SkM are needed to understand the potential functional relevance of the DNA methylation changes observed.

The findings of this preliminary study suggest differences in the DNA methylation profiles in the skeletal muscle of older adults with HIV and uninfected controls of similar chronologic age. The lack of overlap of genes and potential pathways associated with differential methylation in response to training may indicate alternative mechanisms underpinning exercise adaptations in the presence of chronic HIV infection. In‐depth genomic and transcriptomic analyses are needed to better understand the relationships of methylated gene regions to biological pathways and ultimately the function of aging skeletal muscle.

## AUTHOR CONTRIBUTIONS

Catherine M. Jankowski, Colleen G. Julian, and Kristine M. Erlandson involved with conceptualization. Colleen G. Julian and Iain R. Konigsberg involved in methodology. Catherine M. Jankowski, Colleen G. Julian, Kristine M. Erlandson, Melissa P. Wilson, and Iain R. Konigsberg involved in formal analysis and investigation. Catherine M. Jankowski, Colleen G. Julian, Kristine M. Erlandson, Melissa P. Wilson, Iain R. Konigsberg, Jing Sun, and Todd T. Brown involved in writing—original draft preparation. Catherine M. Jankowski, Colleen G. Julian, and Kristine M. Erlandson involved in funding acquisition and resources.

## FUNDING INFORMATION

KME has consulted for ViiV, Merck, and Gilead Sciences, and received research funding from Gilead Sciences, all paid to the University of Colorado. TTB has served as a consultant to Merck, Gilead, ViiV Healthcare, Janssen, and Theratechnologies. Financial support for this research was provided by grants from NCATS Colorado CTSA (UL1TR002535), National Institute on Aging (K23AG050260) to KME, and the National Institute of Diabetes, Digestive, and Kidney Disorders (DK048520). CGJ has received funding from the National Institutes of Health (R01 HD088590, R01 HL138181, R21TW010797). TTB is supported in part by K24 AI120834.

## CONFLICT OF INTEREST STATEMENT

The authors have not and do not currently have a commercial or other association that might pose of conflict of interest with the research.

## Supporting information


Table S1

## Data Availability

Data available upon request from the authors.
